# Self-sampling for human papillomavirus DNA detection: a preliminary study of compliance and feasibility in BOLIVIA

**DOI:** 10.1186/s12905-017-0490-z

**Published:** 2017-12-22

**Authors:** Pedro Surriabre, Gustavo Allende, Marcela Prado, Leyddy Cáceres, Diego Bellot, Andrea Torrico, Karina Ustariz, Shirley Rojas, Jaime Barriga, Pamela Calle, Ligia Villarroel, Rosse Mary Yañez, Marc Baay, Patricia Rodriguez, Véronique Fontaine

**Affiliations:** 1 Laboratorio de Virología, Facultad de Medicina, Universidad Mayor de San Simón, Cochabamba, Bolivia; 2 Unité de Microbiologie Pharmaceutique et Hygiène, Faculté de Pharmacie, Université Libre de Bruxelles, Brussels, Belgium; 3 Hospital Materno Infantil Germán Urquidi, Servicio de Ginecología, Cochabamba, Bolivia; 4 Marie Stopes International, Servicio de Ginecología, Cochabamba, Bolivia; 5 Laboratory of Cancer Research and Clinical Oncology, University of Antwerpen, Wilrijk, Belgium

**Keywords:** Human papillomavirus, HPV screening, Self-sampling, Devices, Acceptability

## Abstract

**Background:**

Cervical cancer incidence and mortality rates in Bolivia are among the highest in Latin America. This investigation aims to evaluate the possibility of using simple devices, e.g. a cotton swab and a glass slide, for self-sampling in order to detect human papillomavirus (HPV) DNA by PCR in cervico-vaginal cells.

**Methods:**

In the first phase of our study we evaluated the use of a glass slide as a transport medium for cervical cells. A physician took paired-cervical samples from 235 women. One sample was transported in Easyfix® solution and the other sample was smeared over a glass slide. Both were further analyzed and compared for human DNA recovery and HPV detection. A kappa value was determined to evaluate the agreement between the HPV DNA detection rates.

In the second phase of the study, 222 women from the urban, peri-urban and rural regions of Cochabamba were requested to perform self-sampling using the following devices: a cotton swab combined with a glass slide, and a vaginal tampon. Women gave their opinion about the self-sampling technique.

Finally, the agreement for high risk-HPV detection between self- and physician-collected samples was performed in 201 samples in order to evaluate the self-sampling technique.

**Results:**

Firstly, the comparison between Easyfix® solution and the glass slide to transport clinical samples gave a good agreement for HPV DNA detection (κ = 0.71, 95% CI 0.60–0.81). Secondly, self-sampling, especially with cotton swab combined with glass slide, would generally be preferred over clinician sampling for a screening program based on HPV detection. Finally, we showed a good agreement between self- and physician collected samples for high risk-HPV detection (κ = 0.71, 95% CI 0.55–0.88).

**Conclusions:**

Simple devices such as a cotton swab and a glass slide can be used to perform self-sampling and HPV DNA detection. Furthermore, most Bolivian women preferred self-sampling over clinician-sampling for cervical cancer screening.

**Electronic supplementary material:**

The online version of this article (10.1186/s12905-017-0490-z) contains supplementary material, which is available to authorized users.

## Background

The incidence and mortality rates of cervical cancer in Bolivia are the highest in Latin America: 39.5 and 16.5 per 100,000 women, respectively [1]. This is particularly noteworthy as cervical cancer can be prevented by opportune screening and treatment of precancerous lesions. Papanicolaou screening rates reported in Bolivia are a matter of concern since it varies from 9 to 28% [2]. Furthermore, rural women are screened less frequently than urban women (18.7% vs. 32.2%) [3]. Moreover, 50% to 80% of screened women are lost during follow-up and the lowest coverage rates are observed in the lowest-income population who are at highest risk for cervical cancer [4].

The current national program for cervical cancer prevention stipulates that every woman should have a Pap test every three years after two consecutive annual negative smears [5]. However, the cytology-based screening program implemented in Bolivia has important problems with long delays and lost screening results [4]. Additionally, the socio-cultural characteristics of the local population and the poor knowledge about HPV infection, especially in the rural regions, might represent an obstacle for women to participate in the current screening program [3–5]. At the same time, very few studies have been carried out in Bolivia to show HPV prevalence and genotype distribution [6]. The ICO information center indicates an approximated prevalence of HPV16 and/or HPV18 of 5.8% in women with normal cytology in Bolivia [2].

It has been well established that a persistent infection by high risk HPV genotypes (hr-HPV) is the most important risk factor for developing cervical cancer [7]. HPV tests are efficient to detect precancerous lesions (CIN2+) and therefore, have been proposed as a primary screening tool for cervical cancer prevention [8]. Additionally, hr-HPV DNA tests can be performed on self-collected cervico-vaginal cells, using appropriate devices. The self-sampling technique offers an interesting possibility to increase participation rates in low-resource settings [9]. Indeed, it has been shown that self-sampling aids to circumvent socio-cultural barriers as demonstrated in other Latin American countries like Argentina [10], México [11] and Nicaragua [12].

Lately, companies have made specialized brushes in order to introduce self-sampling e.g. the Viba-Brush and the Evalyn Brush (Rovers Medical Devices B.V., Oss, The Netherlands) [13, 14]. These brushes, although effective, are expensive to import into Bolivia. Simpler and lower-cost devices have been tested to self-collect cervico-vaginal cells with general good results, such as cotton swabs [15–17], vaginal tampons [18–20] and lavages [21, 22]. Alternative media to transport and store cervical samples have also been tested, e.g. paper filter [21, 23, 24], pads [25], and even mouthwash solution [26].

It is noteworthy that in order to be sustainable in a developing country’s health program, such as in Bolivia, new cost-effective techniques for cervical cell transport should be developed and adapted to the socio-economic characteristics of the population. Glass slides are currently used to transport cervical samples for standard cytology tests and could represent an interesting option to economically and conveniently transport cervical cells for hr-HPV DNA detection. However, this device has not yet been tested to transport samples for detection of HPV DNA.

Our main objective was to evaluate the possibility of introducing self-sampling for HPV DNA detection using simple devices through an acceptability and feasibility study. The first phase of this study was to evaluate the efficiency of a glass slide as an alternative transport medium for cervico-vaginal cells. Secondly, we evaluated women preference for a self-sampling device between a cotton swab combined with a glass slide and a vaginal tampon, in the urban, peri-urban and rural areas of Cochabamba. Finally, we compared self- and physician-collected samples for hr-HPV detection to assess our self-sampling technique.

## Methods

### Study populations and cervical sample collection

The Bio-ethical Committee of the “Universidad Mayor de San Simón” approved the study protocol (October 30th, 2014) and each participant signed an inform consent form before enrollment.

For the first phase of the study, a group of 235 women attending gynecological services for cytological abnormalities were recruited in the “Hospital Materno Infantil Germán Urquidi” and the gynecological outpatient clinic of the Non-Governmental Organization “Marie Stopes International” in the city of Cochabamba. A physician collected paired-cervical samples from each woman. One sample was placed in Easyfix® solution and the second sample was smeared on a glass slide. Cervical samples were collected in alternate order to avoid bias.

For the second phase of the study, a group of 222 women between 25 and 59 years old from three different regions (urban, peri-urban and rural areas) tested 2 self-sampling devices: a cotton swab and a vaginal tampon. The recruitment was done through women’s organizations in all areas of the study. The leader of the organization called a meeting and all women who were interested to participate in the study signed an inform consent. No previous cytological results were required to be included in this study. Women were explained how to perform self-sampling with both devices through images (see Additional file 1) and a video. Self-sampling with the cotton swab was performed first to avoid cell depletion with the vaginal tampon. Briefly, women were asked to introduce a cotton swab deep inside the vaginal tract until bottom was reached, to rotate the swab 3 times, to take it out and then to smear the cells once over the glass slide. Each slide should be put inside a small cardboard box specifically designed for it. The box is then transported in a zippered storage bag to the laboratory. To perform self-sampling with tampon, women were asked to introduce it deep inside the vagina for at least 30 s, then to remove it and to place it in a Falcon tube containing 15 ml of Easyfix® solution. Women were asked to complete a questionnaire to evaluate their experience with self-sampling and their preference for a device.

Finally, hr-HPV detection was performed in self- and physician-collected samples from the same patients in a group of 201 women. Self-sampling was always performed before physician sampling. Women first performed self-sampling with cotton swabs and smeared cervical cells on glass slides, as previously described. Additionally, physician collected samples from the same patients using cervical brushes and cervical cells were also smeared on glass slides.

### DNA preparation

DNA extraction from all samples was always performed within 2 weeks after they were collected. Total cellular DNA from cervical samples conserved in Easyfix® solution was extracted as follows: 1 ml of cell sample was centrifuged (3000 x g for 10 min) and washed with 300 μl of phosphate-buffered saline solution, centrifuged again (3000 x g for 10 min), and re-suspended in 150 μl of PK-1 buffer (10 mM Tris-HCl pH 7.4, 50 mM KCl, 0.45% NP-40, 0.45% Tween 20, 0.1 mg/ml gelatin, 100 μg/ml proteinase K). The cell suspension was incubated for 16–20 h. at 56 °C and then incubated at 100 °C for 10 min for proteinase K inactivation. Cell debris was removed by centrifugation (14,000 rpm for 30 s). A quality control of DNA extraction was performed by a PCR using the primers PC04 (5’-CAACTTCATCCACGTTCACC-3’) and GH20 (5’-GAAGAGCCAAGGACAGGTAC-3’), amplifying a 260 bp fragment from the human β-globin gene [27, 28]. Only samples giving a positive result in the β-globin PCR were further analyzed for HPV DNA detection.

For samples transported on a glass slide, DNA was extracted as follows: cells were detached from the glass slide surface with a micropipette tip using 300 μl of Tris-HCl 10 mM pH 8.3 solution. Cells were then centrifuged (3000 x g for 10 min), and DNA was extracted from the cell pellets as described above, in 150 μl of PK-1 buffer.

Cervical samples collected with the tampon and transported in 15 ml Easyfix solution were treated as follows: The tampon was taken out from the Falcon tube and squeezed with a syringe piston to recover the cells. The solution was centrifuged as described above and DNA extraction was performed as previously described for the cells stored in Easyfix® solution.

### HPV DNA detection

HPV DNA was detected with a consensus PCR using the primers GP5+ (5’-TTTGTTACTGTGGTAGATACTAC-3’) and GP6+ (5’-GAAAAATAAACTGTAAATCATATTC-3’), amplifying a 150 bp fragment from the L1 region of the HPV genome [29]. PCR reactions were carried out in 25 μl of a PCR mixture containing 3.5 mM MgCl_2_, 0.2 mM dNTP mix, 1 μM of each primer, 1 U of GoTaq® G2 Hot Start Polymerase (Promega, USA) and 5 μl of crude DNA extract. PCR was performed in a T100™ Thermal Cycler (Bio-Rad, USA). Amplified fragments were visualized in a 2% agarose gel (Sigma-Aldrich, USA) incubated with 0.5 μg/ml ethidium bromide solution (Sigma-Aldrich). Detection of hr-HPV DNA was performed using an enzyme-linked immunoassay as described by Jacobs et al. [30] . Oligo-probes for detection of 12 h-HPV genotypes were used (16, 18, 31, 33, 35, 39, 45, 51, 52, 56, 58, 59).

### Statistics

Statistical analyses were performed using SPSS software. Cohen’s kappa factor was calculated to evaluate the agreement between HPV detection results [31]. Kappa values of <0.20, 0.21 to 0.40, 0.41 to 0.60, 0.61 to 0.80 and 0.81 to 1.00 were considered poor, fair, moderate, good and very good agreement, respectively [32].

## Results

### Evaluation of a glass slide as transport medium for cervico-vaginal samples

To evaluate the possibility of using a glass slide to transport cervical cells samples for HPV DNA detection through PCR, we recruited 235 women with cytological abnormalities and compared human β-globin and HPV PCR results from their samples. The PCR results gave similar detection rates of human DNA (94% in Easyfix® solutions vs. 91% on glass slides) and HPV DNA (30% in Easyfix® solutions vs. 33% on glass slides). Comparison of HPV detection rates obtained for the samples with positive β-globin PCR (207 cases) showed a good level of concordance (κ = 0.71, 95% CI 0.60–0.81, Table 1).

**Table 1:** Comparison of the HPV DNA detection results obtained in samples transported in Easyfix® solutions and on glass slides

Easyfix®	Glass Slide		Total
	Positive	Negative	
Positive	55	11	66
Negative	16	125	141
Total	71	136	207

Kappa (κ) = 0.71 (95% CI, 0.60–0.81)

### Assessment of women’s preference for a self-sampling device

This study population included 222 women from three regions of Cochabamba: urban (*N* = 96), peri-urban (*N* = 66) and rural (*N* = 60). Considerable differences were observed between urban and rural populations in the education level. Most urban women (66%) have been to the university while most rural women (82%) only attended primary school. Furthermore, 76% of women reported to ever had a Pap test (data not shown).

In general, this study population indicated that they would prefer self-sampling over clinician-sampling for a future cervical cancer screening based on HPV DNA detection (64% vs. 14%, Table 2). Concerning the sampling device, most women thought that the cotton-swab was easier (77% vs. 59%) and more comfortable (80% vs. 56%) to use than the vaginal tampon. These data are summarized in Table 3. Self-collected samples using the cotton swabs and the vaginal tampons were analyzed for the presence of human DNA (β-globin PCR) and HPV DNA (GP5+/6+ PCR). Human DNA was found in 91% of samples collected with the cotton swab and in only 77% of samples collected with the tampon. A GP5+/6+ PCR was performed only in samples with a β-globin positive result. HPV detection rates in samples collected with cotton swab and tampon were 15% and 11% respectively (Table 4).

**Table 2:** Women’s appreciation and confidence about self-sampling

No. (%) of women answering the corresponding statement					
Region	N	Which kind of sampling method would you prefer for a cervical cancer screening program?			
		Physician	Self-sampling	No particular preference	NA^a^
Urban	96 (100)	14 (15)	62 (65)	7 (7)	13 (13)
Peri-urban	66 (100)	14 (21)	39 (59)	5 (8)	8 (12)
Rural	60 (100)	3 (5)	42 (70)	15 (25)	0 (0)
Total	222 (100)	31 (14)	143 (64)	27 (12)	21 (10)

^a^No answer

**Table 3:** Acceptability for a self-sampling device

No. (%) of women who answered the corresponding statement													
Region	N	Was self-sampling easy to perform?						Was self-sampling comfortable to perform?					
		With cotton swab?			With vaginal tampon?			With cotton swab?			With vaginal tampon?		
		Yes	No	NA^a^	Yes	No	NA^a^	Yes	No	NA^a^	Yes	No	NA^a^
Urban	96 (100)	70 (73)	10 (10)	16 (17)	57 (59)	19 (20)	20 (21)	76 (79)	6 (6)	14 (15)	54 (56)	17 (18)	25 (26)
Peri-urban	66 (100)	52 (79)	6 (9)	8 (12)	37 (56)	18 (27)	11 (17)	53 (80)	4 (6)	9 (14)	30 (45)	21 (32)	15 (23)
Rural	60 (100)	49 (82)	6 (10)	5 (8)	38 (63)	13 (22)	9 (15)	48 (80)	6 (10)	6 (10)	42 (70)	10 (17)	8 (13)
Total	222 (100)	171 (77)	22 (10)	29 (13)	132 (59)	50 (23)	40 (18)	177 (80)	16 (7)	29 (13)	126 (56)	48 (22)	48 (22)

^a^No answer

**Table 4:** Human (β-globin PCR) and HPV DNA (GP5+/6+ PCR) detection rates in self-collected samples with a cotton swab and a vaginal tampon

PCR	Cotton swab			Vaginal tampon		
	Positive	Negative	Total	Positive	Negative	Total
β-globin	203 (91)	19 (9)	222 (100)	172 (77)	50 (23)	222 (100)
GP5+/6+	31 (15)	172 (85)	203 (100)	19 (11)	153 (89)	172 (100)

### Comparison between self- and physician-collected samples for hr-HPV DNA detection

This comparison was performed in a group of 201 women who self-collected samples with a cotton swab. The samples collected by the physician were obtained with a cytological brush. In both sampling methods, the collected cells were smeared over a glass slide. The agreement for hr-HPV DNA detection between these two collection methods was good (κ = 0.71, 95% CI 0.55–0.88, Table 5).

**Table 5:** Comparison of the hr-HPV DNA detection results obtained in self- and physician collected samples

Self-sampling	Physician		Total
	Positive	Negative	
Positive	16	6	22
Negative	5	174	179
Total	21	180	201

Kappa (κ) = 0.71 (95% CI, 0.55–0.88)

## Discussion

The main objective of this study was to evaluate the possibility of introducing self-sampling for HPV DNA detection using simple and cheap devices in Cochabamba, Bolivia. The economic situation in Bolivia requires the use of materials and techniques that would allow performing a cheap, yet robust HPV DNA test.

Self-sampling devices are not commercially available in Bolivia since HPV detection is not perform in a routine basis for cervical cancer prevention. For that reason, we decided to use simple and cheap devices for self-collection of cervico-vaginal samples and to transport them. In that sense, we thought on two possibilities. The first one was to use a cotton swab to collect the cells sample and then to use a glass slide to transport them. It has been shown previously that self-sampling with a simple cotton swab had a similar sensitivity for detection of hr-HPV infections compared to liquid based cytology [16]. However, the glass slide had never been tested before to transport cervico-vaginal samples for HPV detection. Our second possibility for a self-sampling device was a vaginal tampon, which can be used alone to collect and to transport the cells sample. Indeed, comparative studies with physician-collected samples have shown that the vaginal tampon is also a good device to collect cervico-vaginal cells [15, 19, 20]. Moreover, it has been shown that the quality of samples collected with vaginal tampons was not affected by the menstrual cycle [33].

Before evaluating self-sampling with these simple devices in the women population, we had to be sure that the glass slide was an efficient medium to transport the cells. To do so, we first compared the quality of samples transported on the glass slide to samples transported in Easyfix® fixative fluid, which was previously shown to be an adequate medium to conserve cervical cells [34, 35]. The detection rates of human and viral DNA were similar in samples transported in both conditions. This implies that glass slide is a convenient and an adequate medium for cell transport. However, it is still possible to increase DNA recovery in samples transported on glass slides by improving the DNA extraction protocol. Based on our results with the glass slide, we felt sure to combine the cotton swab and the glass slide for self-collection and transport of the samples and to offer this possibility to the women population. To our knowledge, this is the first study proving the effectiveness of the dry collection, storage and transport of cervical cells on a glass slide for HPV DNA detection.

In the second phase of our study we proposed self-sampling with these simple devices: a cotton swab combined with a glass slide and a vaginal tampon to our women population. The results showed that women preferred the cotton swab to the vaginal tampon in terms of easiness and comfort. Furthermore, the detection rates of human and viral DNA were much better in samples collected with the cotton swab than in samples collected with the tampon. One possible explanation is the fact that 72% of women in our study population (data not shown) never used before a vaginal tampon. This implies that women could have misused the device even though 59% of them reported that self-sampling with the tampon was easy. This difference in DNA recovery could also be due to the sampling procedure, i.e. in our study women introduced the vaginal tampons for maximum 30 s, probably allowing little time for the cells to be collected by the vaginal tampon. Indeed, a previous study has shown that tampon samplings with longer cell exposure were equivalent to two swabs for detection of hr-HPV genotypes [18].

The use of a cotton swab is not more common than a vaginal tampon among Bolivian women, however it seems that the procedure for self-collection with the swab is easier to understand for them. It is worth noting that a previous study assessed the possibility of using a cotton swab for self-sampling and concluded that it is not a safe method for sample collection [36]. The differences in results may be explained by different protocols for sample handling, transport, and DNA extraction.

Another important finding in our study is that most women would prefer self-sampling over standard clinician-sampling for a possible screening program based on HPV detection in all three regions (urban, peri-urban and rural areas). This is particularly important considering the socio-cultural differences between this regions, specially among urban and rural populations. Furthermore, as 76% of the surveyed women reported having a previous Pap test, they can really compare between sampling procedures. Our results reflect Bolivian women’s commitment to perform self-sampling on a routine basis since more than 80% of them (data not shown) feel confident about the efficiency of the self-collection and therefore would prefer it in a primary screening program for cervical cancer prevention. A recent study about self-sampling acceptability was carried-out in a population with similar socio-cultural characteristics in the northern region of Argentina. This study also found a high percentage of acceptability (86%) [10].

As a final step in our study, we evaluated the efficacy of self-sampling for the detection of hr-HPV detection by comparing it with the standard clinician-sampling. In the design of this part of the study we decided that the physician would use the glass slide to transport the cells samples since we validated its use for that purpose in the first phase of our study. Our results indicate a good agreement between the sampling methods and suggest that the self-collection using a simple cotton swab with a glass slide is a valid method for HPV detection. A previous study has also shown good agreement in hr-HPV detection between clinician and self-sampling with a cotton swab [37].

The results obtained in this study prompted us to design and prepare our own self-sampling kit to offer to the women population. This kit consists in a zipper storage bag containing a cotton swab, a glass slide (properly covered in a cardboard box) and a pair of gloves. The cost of this kit is around half of a dollar, which represents an accessible price for the government considering a future population screening program based on self-sampling.

## Conclusions

We evaluated the possibility of introducing self-sampling in Bolivia using simple and cheap devices such as cotton swab and glass slide. We showed that these devices can be used to collect and transport cells with acceptable rates for human and viral DNA detection. Moreover, according to the results of our questionnaire, women would prefer self-sampling for a screening program based on HPV DNA detection. Nevertheless, further studies should be performed to prove that our self-sampling method has a sufficient clinical sensitivity for detection of precancerous lesions (CIN2+).

## Additional file

Additional file 1: Biptico 1 Information provided to patients, recto page of folder. Biptico 2 Information provided to patients, verso page of folder. Triptico 1 Information provided to health centers and patients, recto page of folder. Triptico 2 Information provided to health centers and patients, verso page of folder. Some images from http://www.msal.gob.ar/images/stories/ryc/graficos/0000000773cnt-20-INSTRUCTIVO-autotoma.pdf have been adapted. (ZIP 386 kb)

## Abbreviations

CIN2+: Cervical intraepithelial neoplasia grade 2 or worse
DNA: Deoxyribonucleotide acid
HPV: Human papillomavirus
hr-HPV: high-risk human papillomavirus
ICO: Institut Catala d’Oncologia (Catalan Institute of Oncology)
PCR: Polymerase chain reaction
