# Supplementary figures and images for: Self-sampling for human papillomavirus DNA detection: a preliminary study of compliance and feasibility in BOLIVIA
Source: BMC Womens Health. 2017 Dec 22;17:135. doi: 10.1186/s12905-017-0490-z (PMC5741906; doi:10.1186/s12905-017-0490-z)

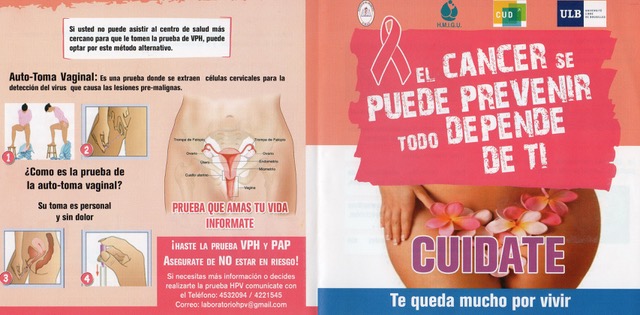

Supplement: Additional file 1: — Biptico 1 Information provided to patients, recto page of folder. Biptico 2 Information provided to patients, verso page of folder. Triptico 1 Information provided to health centers and patients, recto page of folder. Triptico 2 Information provided to health centers and patients, verso page of folder. Some images from http://www.msal.gob.ar/images/stories/ryc/graficos/0000000773cnt-20-INSTRUCTIVO-autotoma.pdf have been adapted. (ZIP 386 kb) [file 12905_2017_490_MOESM1_ESM.zip › Biptico 1R4.jpeg]

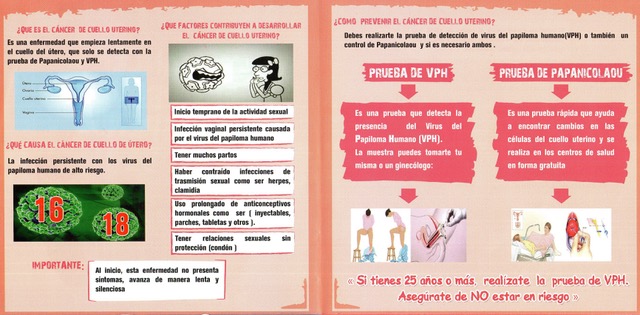

Supplement: Additional file 1: — Biptico 1 Information provided to patients, recto page of folder. Biptico 2 Information provided to patients, verso page of folder. Triptico 1 Information provided to health centers and patients, recto page of folder. Triptico 2 Information provided to health centers and patients, verso page of folder. Some images from http://www.msal.gob.ar/images/stories/ryc/graficos/0000000773cnt-20-INSTRUCTIVO-autotoma.pdf have been adapted. (ZIP 386 kb) [file 12905_2017_490_MOESM1_ESM.zip › Biptico 2R4.jpeg]

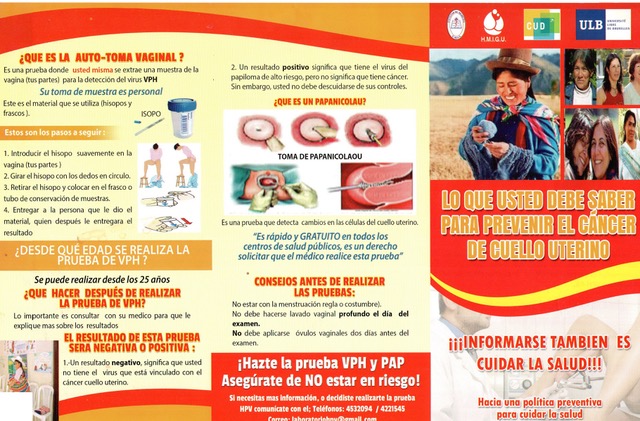

Supplement: Additional file 1: — Biptico 1 Information provided to patients, recto page of folder. Biptico 2 Information provided to patients, verso page of folder. Triptico 1 Information provided to health centers and patients, recto page of folder. Triptico 2 Information provided to health centers and patients, verso page of folder. Some images from http://www.msal.gob.ar/images/stories/ryc/graficos/0000000773cnt-20-INSTRUCTIVO-autotoma.pdf have been adapted. (ZIP 386 kb) [file 12905_2017_490_MOESM1_ESM.zip › Triptico 1R4.jpeg]

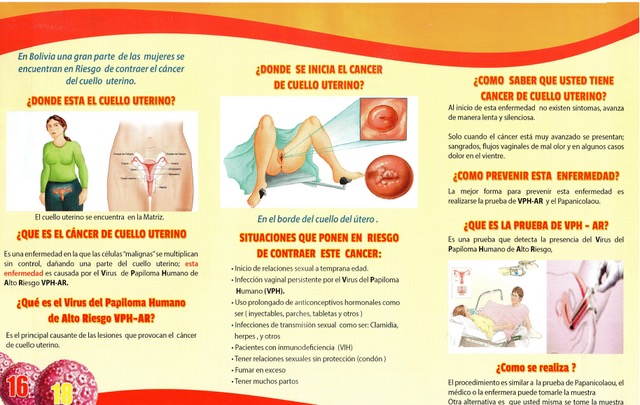

Supplement: Additional file 1: — Biptico 1 Information provided to patients, recto page of folder. Biptico 2 Information provided to patients, verso page of folder. Triptico 1 Information provided to health centers and patients, recto page of folder. Triptico 2 Information provided to health centers and patients, verso page of folder. Some images from http://www.msal.gob.ar/images/stories/ryc/graficos/0000000773cnt-20-INSTRUCTIVO-autotoma.pdf have been adapted. (ZIP 386 kb) [file 12905_2017_490_MOESM1_ESM.zip › Triptico 2R4.jpeg]
